# Toward Equitable Kidney Care: Insights from the Survey Among Polish Doctors on the Women’s Health in Chronic Kidney Disease Management

**DOI:** 10.3390/jcm15010196

**Published:** 2025-12-26

**Authors:** Weronika Przybyszewska, Karol Gawalski, Barbara Bijak, Aleksandra Rymarz, Jolanta Małyszko

**Affiliations:** 1 Department of Nephrology, Dialysis and Internal Medicine, Medical University of Warsaw, 02-097 Warsaw, Poland; weronika.przybyszewska@wum.edu.pl (W.P.); s083707@student.wum.edu.pl (B.B.); aleksandra.rymarz@wum.edu.pl (A.R.); 2 Doctoral School, Medical University of Warsaw, 02-097 Warsaw, Poland; karol.gawalski@wum.edu.pl; 3 Department of Pathology, Military Institute of Medicine, 04-141 Warsaw, Poland; 4 Laboratory of Experimental Medicine, Medical University of Warsaw, 02-097 Warsaw, Poland

**Keywords:** chronic kidney disease, nephrology care, women’s health, nephrology education, pregnancy and CKD, gender-related health problems

## Abstract

**Background/Objectives:** Chronic kidney disease is more prevalent among women, and there are significant disparities in the management of female patients. Our study aimed to assess the clinical experience and educational needs of physicians in the treatment of reproductive and sex and gender-related health problems in patients with CKD. **Methods:** The three-part survey was distributed among nephrologists and other internal medicine specialists in Poland, both online and in paper form. **Results:** A total of 116 physicians participated in the survey, including 81 nephrologists. Most respondents (64.7%) were female and practiced in multispecialty hospitals (72.4%). While 97.4% managed patients with CKD, only 37.9% reported caring for pregnant women. Experience in sex-specific and pregnancy-related issues was limited—56.9% reported low or minimal experience in managing CKD during pregnancy. Residency training lacked coverage of crucial topics, with 66.2% reporting limited teaching on sex-related CKD progression. Over 90% supported integrating reproductive planning and interdisciplinary care into nephrology, favoring guidelines, conferences, and webinars for education. **Conclusions:** Our study has highlighted a critical discrepancy between the importance of sex-specific and reproductive health considerations in the management of CKD and the current level of clinical experience among Polish doctors.

## 1. Introduction

Over the last decades, we have been observing a significant increase of about 29.3% in the prevalence and 41.5% in mortality of chronic kidney disease (CKD) [1]. May 2025 marks an important milestone for the entire nephrology society as the WHO (World Health Organization) prioritized CKD within the WHO’s noncommunicable diseases (NCD) agenda [2,3]. This historic declaration raises awareness about the burden of kidney diseases, promotes early detection, and motivates actions to guarantee equitable access to care for patients in all communities [4].

According to epidemiological data, CKD is more prevalent among women than men [5]. However, compared to male patients, women are more likely to be offered dialysis at lower estimated glomerular filtration rate (eGFR) levels, are less frequently included on the transplantation lists, and report a lower perceived quality of life. While some of these disparities may be attributed to biological factors, a significant portion stems from diverse socioeconomic influences [6].

As indicated in the Healthy Life in an Urban Setting (HELIUS) study, those biological factors have been widely investigated; however, the impact of sociocultural factors still remains scarce. The research group has found specific gender-related factors that significantly increase the risk of developing kidney diseases. CKD was more common in all women who did little housework, worked part-time or were unemployed, and among men whose financial contribution was equal to their partners’ or who were unemployed. This risk could not be explained by other well-known factors contributing to the development of CKD [7]. Nephrologists also increasingly acknowledge the impact of gender disparities in CKD and often perceive women as disadvantaged when faced with kidney disorders. This stems from multiple barriers, including limited access to equitable care, the burden of fulfilling traditional gender roles, and extensive caregiving responsibilities. Women are more vigilant and proactive regarding their health compared to men, which leads to better management of their disease. However, due to many domestic and family-related obligations, as well as concerns about their physical appearance, the time of the initiation of dialysis tends to be delayed compared to male patients [8].

Women at different life stages require a tailored approach to disease management. This need has already been increasingly acknowledged in other medical specialties. For instance, it has also been reflected in the recent Polish hypertension guidelines, which emphasize sex-specific considerations in the management of this chronic disease [9]. Although women’s health issues are gaining visibility within the nephrology community [10], there is still a lot more to do to provide equitable care for all female patients. Notably, despite the growing number of breakthrough clinical trials in nephrology, women continue to be underrepresented in clinical trials compared to their actual prevalence of CKD [11]. This may even lead to further implications and greater risk of developing adverse side effects as medication dosing is generally investigated on the groups mostly represented by men [12].

In light of all these data, our study aimed to assess physicians’ clinical experience, confidence, and educational needs in managing sex-specific and reproductive health issues in patients with CKD. By surveying doctors across diverse workplace settings, we sought to identify gaps in training and practice, and to explore opportunities for integrating women’s health more effectively into nephrology care.

## 2. Materials and Methods

The study was designed to include both residents and specialists of nephrology and from other fields of internal medicine. The questionnaire was developed following a literature review and subsequently refined through collaborative discussions among the authors. The study has received a positive approval from the Bioethics Committee of the Medical University of Warsaw. The complete survey questionnaire is available in the Supplementary Materials.

The survey consisted of three sections. The first gathered basic demographic information, including gender, years of clinical experience, medical specialty, and workplace setting. To better understand the scope of each respondent’s patient population, additional questions addressed average patient age, gender distribution, and employment status.

Section 2 concerned the clinical experience of the participants in the management of patients with CKD, including those undergoing dialysis. Their level of clinical experience was assessed using a 5-point Likert scale—from 1 (very low experience), 2 (low experience), 3 (moderate experience), 4 (high experience), to 5 (very high experience).

Further items explored the frequency, using a four-point Likert scale (never, rarely, often, always), with which physicians address women’s and reproductive health issues during the process of taking medical history. The survey also investigated the use of ACE-I/ARBs in clinical practice, including prescribing indications and significant reproductive health considerations such as their teratogenic effect.

Section 5 of the survey was directed exclusively to nephrologists, both trainees and certified. A series of Likert scale items was used to evaluate whether sex-specific issues in CKD management (the influence of gender-related factors on disease progression, the impact of CKD, dialysis, and immunosuppressive therapy on fertility, and the management of kidney disorders during pregnancy) are adequately addressed in residency training programs.

Respondents were then asked to self-assess their clinical experience and knowledge across several domains, using a Likert scale from 1 (very low experience), 2 (low experience), 3 (moderate experience), 4 (high experience), to 5 (very high experience). Topics included pregnancy planning in CKD patients and the use of ART in this population.

The survey also gathered nephrologists’ views on the need for sex-specific clinical guidelines, the importance of tailoring immunosuppressive therapy to reproductive plans, and the value of interdisciplinary collaboration, including the establishment of referral centers for pregnant patients with CKD. These opinions were measured using a four-point Likert scale (strongly disagree, somewhat disagree, somewhat agree, strongly agree).

Finally, participants were asked to indicate their preferred activities for further education on women’s health in nephrology, such as conferences, structured courses, webinars, or clinical guidelines issued by professional societies.

The survey was distributed using a voluntary, anonymous, convenience sampling strategy. The questionnaire was distributed in both paper and digital formats. Printed copies were delivered directly to physicians working in internal medicine and nephrology wards in Warsaw and other hospitals across Poland. The online version, prepared using the Google Forms platform, was disseminated via social media groups dedicated to medical practitioners and circulated through the mailing list of the Polish Society of Nephrology. Because the online distribution channels were open, an exact response rate could not be calculated. Data collection took place between March and July 2025.

Statistical analysis was performed using Statistica software (v13.3, StatSoft, Tulsa, OK, USA). Data expressed on a qualitative scale were presented as the number and percentage. Descriptive statistics were used to summarize demographic characteristics and response distributions. Exploratory subgroup analyses were conducted using the Chi-squared test or Chi-square test with Yates’ correction to compare the distributions of these parameters between groups defined by specialty, years of experience, stage of training, and workplace setting. Results were considered statistically significant when *p* < 0.05.

The Microsoft 365 Copilot was used for data analysis and text editing during the preparation of this study.

## 3. Results

### 3.1. General Part of the Survey for All Specialists

#### 3.1.1. Demographical Data and Patients’ Profile

A total of 116 physicians participated in the survey, including 81 nephrologists, of whom 68 (84%) were certified specialists, and 13 (16%) were trainee doctors. Female respondents comprised the majority, accounting for 64.7% (n = 75) of the sample, while male participants represented 35.3% (n = 41).

Among all respondents, 76,7% (n = 89) held specialist-level qualifications and nearly 70% (n = 81) reported over 10 years of clinical experience. Residents constituted 23.3% (n = 27) of the sample. In terms of specialization, 81 nephrologists took part in the survey, and 79 reported a background in internal medicine. Other declared specialties included family medicine (n = 4; 3.5%), cardiology (n = 3; 2.6%), and diabetology (n = 1; 0.9%).

The most common workplace setting was a multispecialty hospital (n = 84; 72.4%), followed by specialist outpatient clinics (n = 41; 35.3%) and private healthcare facilities (n = 32; 27.6%). Primary care clinics (n = 16; 3.8%) and municipal or county hospitals (n = 8; 6.9%) were less frequently represented. A detailed summary is presented in Table 1.

Respondents predominantly cared for older adults, with 76.7% (n = 89) indicating that their patient population was primarily aged 60–80 years. Patients were reported to be almost evenly distributed by gender in 56% (n = 65) of cases, while 21.6% (n = 25) of physicians noted a predominance of female patients. Most patients had completed secondary or vocational education (n = 98; 84.5%), and the vast majority were retired or receiving disability pensions (n = 94; 81.0%).

The vast majority of respondents (97.4%, n = 113) reported managing patients diagnosed with CKD. Among them, 60.3% (n = 70) estimated that over half of their patient population had CKD, while 26.7% (n = 31) reported a prevalence between 10–50%. Only 10.3% (n = 12) indicated that fewer than 10% of their patients were affected.

A total of 98 physicians (84.5%) reported caring for patients undergoing dialysis. Among patients, hemodialysis was the predominant modality (n = 85; 73.3%), while peritoneal dialysis was reported by only 3.4% of respondents (n = 4).

Only 44 respondents (37.9%) reported providing care to pregnant women in their clinical practice. Among those, the estimated annual number of pregnant patients was generally low: 33.6% (n = 39) cared for fewer than 50 such patients per year, and only 4.3% (n = 5) reported managing more than 50. The patients’ profile is summarized in Table 2.

#### 3.1.2. Level of the Clinical Experience in the Management of Patients with CKD Across Various Specialties

Respondents across various specialties reported high levels of clinical experience in managing patients with CKD: 58.6% (n = 68), indicating very high, and 13.8% (n = 16) high experience. Moderate experience was reported by 14.7% (n = 17), while only 12.9% (n = 15) described their experience as low or minimal. The higher values of experience level were more frequently reported by physicians with greater experience compared to those with less than 5 years of experience. This association was statistically significant (*p* = 0.003).

Experience in assessing the impact of sex-related factors on kidney disease development was more evenly distributed. Very high experience was reported by only 17.2% (n = 20), high by 19.8% (n = 23), and moderate by 19.8% (n = 23). Low or minimal experience was reported by around 43.1% (n = 50). Among physicians with >10 years of experience, 48.1% (n = 39) reported high or very high experience, while minimal or low experience was most common among those with <5 years (81%, n = 17). This difference was also statistically significant (*p* = 0.002).

Experience in managing kidney diseases related to pregnancy was generally low. Only 6% (n = 7) reported very high experience, and 14.7% (n = 17) high, while 22.4% (n = 26) indicated moderate experience. Minimal or low experience was reported by the majority of doctors—56.9% (n = 66). The association between the years of practice and experience level in this field was also significant (*p* = 0.004).

Regarding the impact of immunosuppressive therapy on fertility, very high experience was reported by 17.2% (n = 20), high by 16.4% (n = 19), and moderate by 22.4% (n = 26). Minimal or low experience was reported by 43.9% (n = 51). Very high experience was reported by 23.5% (n = 19) of physicians with >10 years of practice, compared to 4.8% (n = 1) among those with <5 years.

Similar analysis was also conducted to assess the relationship between the level of expertise and workplace setting. Respondents working in private healthcare facilities reported significantly higher experience in managing CKD (*p* = 0.021) and in assessing sex-related factors in kidney disease development (*p* = 0.040) than those working in other workplaces. Among those employed in private settings, 75% (n = 24) reported very high experience in CKD management, compared to 52.4% (n = 44) outside this setting. Similarly, 53.1% (n = 17) of private-sector respondents reported high or very high experience in evaluating sex-related factors, compared to 30.9% (n = 26) in other settings.

A statistically significant difference was also observed in experience related to the impact of immunosuppressive therapy on fertility among respondents working in primary care clinics, with lower levels of experience reported overall (*p* = 0.025). None of the respondents working in a primary care setting reported very high experience, compared to 20% (n = 20) in other settings.

No differences were found in the experience related to pregnancy-associated kidney diseases across workplace types, including multispecialty hospitals, district hospitals, specialist outpatient clinics, or private facilities. The summary of this data can be found in Table 3.

#### 3.1.3. Women’s Health-Related Issues in Medical History

**Figure 1:**
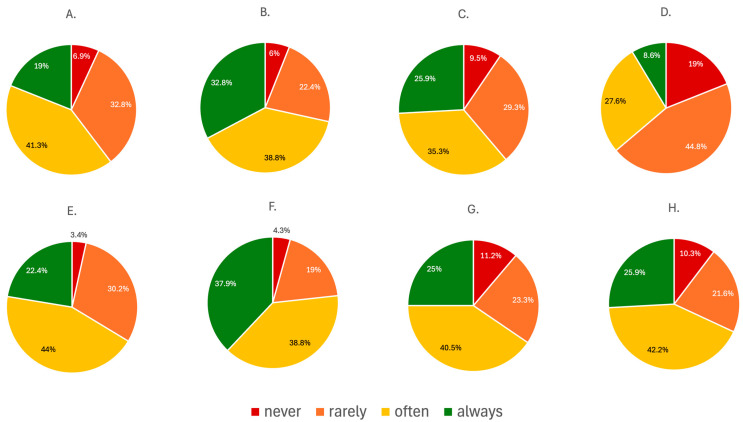
The summary of the responses regarding the frequency of addressing women’s health issues while taking medical history: (**A**) menstrual regularity; (**B**) number of pregnancies; (**C**) number of miscarriages; (**D**) perimenopausal vasomotor symptoms; (**E**) use of contraception; (**F**) the date of the last gynecological visit; (**G**) the date of the last mammography (MMG) and breast ultrasound (USG); (**H**) the date of the last Pap-smear.

The survey revealed considerable variation in the frequency of collecting reproductive health-related information during medical history-taking. Most respondents reported routinely asking about menstrual regularity, with 41.3% (n = 48) doing so often and 19% (n = 22) always, while 32.8% (n = 38) asked rarely and only 6.9% (n = 8) never addressed this topic. Questions regarding pregnancy history were more consistently included, with 71.6% (n = 83) of physicians asking often or always. Similarly, 61.2% (n = 71) inquired about previous miscarriages with regularity, though nearly 40% (n = 45) did so infrequently or not at all. Perimenopausal symptoms were the least frequently addressed topic, with only 8.6% (n = 10) of respondents always asking and 19% (n = 22) never including this topic in routine assessments. Contraceptive use was discussed often or always by 65.4% (n = 77) of physicians, while 30.2% (n = 35) asked rarely. Questions about the timing of the last gynecological visit were common, with 74.7% (n = 89) asking often or always. Breast imaging (MMG/USG) and cervical cytology were also frequently addressed, with 65.5% (n = 76) and 68.1% (n = 79) of respondents, respectively, asking about these screenings regularly. The presented data is summarized in Figure 1.

When asked about reproductive planning, 86.2% (n = 100) of physicians reported discussing procreative intentions with patients of reproductive age, although only half extended this question to both women and men. Most respondents (n = 77; 95.1%) with >10 years of experience reported routinely asking about reproductive plans, from which 51.9% (n = 42) asked only women, while 43.2% (n = 35) asked both women and men. However, 38.1% (n = 8) of respondents with <5 years of experience reported not asking such questions at all. This difference was statistically significant (*p* < 0.001).

In private healthcare facilities, 65.6% (n = 21) of respondents reported asking only women about reproductive plans, compared to 34.5% (n = 29) in other workplace settings. None of the respondents working in the private sector reported omitting this question entirely, while 19.1% (n = 16) of those practicing in other settings did. The association was statistically significant (*p* < 0.001).

A similar pattern was observed in specialist outpatient clinics, where 53.7% (n = 22) of respondents asked only women and only 2.4% (n = 1) omitted this question. This differed significantly from other settings (*p* = 0.010). In multispecialty hospitals, respondents were more likely to ask both women and men (46.4%, n = 39), while 16.7% (n = 14) did not ask at all (*p* = 0.064).

#### 3.1.4. Survey Insights on Angiotensin-Converting Enzyme Inhibitors or Angiotensin II Receptor Blockers ACE-I/ARBs Use

Most respondents (n = 112, 96.6%) reported using ACE-I or ARBs in their clinical practice. Regardless of the number of years of clinical experience, the majority reported using this class of drugs: 95.2% (n = 20) with <5 years, 100.0% (n = 14) with 5–10 years, and 96.3% (n = 78) with >10 years. The difference between these groups was not found to be statistically significant. The use of ACE-I/ARB was also consistent between all workplace settings; the differences between various facilities were not significant.

These medications were most prescribed for either one or for more indications simultaneously, including hypertension (n = 110; 98.2%), nephroprotection (n = 104; 92.9%), and heart failure or cardioprotection (n = 98; 87.5%). Other indications were reported by 10.7% (n = 12) of physicians.

Reproductive health considerations were variably addressed when initiating ACE-I/ARB therapy. While 41.1% (n = 46) of respondents always asked about procreative plans prior to initiation of the treatment, 28.6% (n = 32) did so often, and 23.2% (n = 26) rarely. A small proportion (n = 8; 7.1%) never addressed this issue.

Nearly half of the physicians (n = 57; 50.9%) consistently informed patients about the important teratogenic risks associated with ACE-I/ARB use, while 24.1% (n = 27) did so frequently and 18.7% (n = 21) rarely. Similarly, 45.6% (n = 51) always discussed the need for contraception during treatment, though 30.3% (n = 34) did so infrequently or not at all.

**Figure 2:**
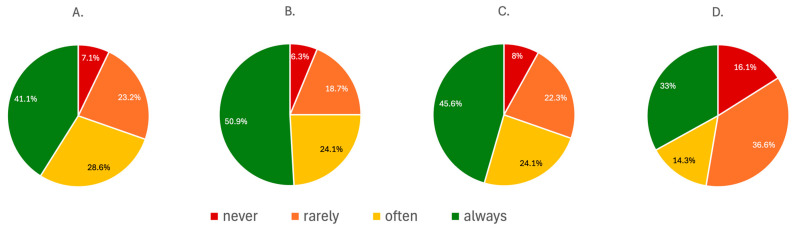
The summary of the responses regarding the frequency of addressing women’s health issues during initiation of ACE-I/ARB therapy: (**A**) discussion about procreative plans of the patient; (**B**) information provided about teratogenic risks; (**C**) counseling on the need for contraceptive use during treatment; (**D**) documentation of teratogenic risk and contraceptive counseling in the medical record.

Documentation of these discussions in medical records was inconsistent among respondents. Only 33% (n = 37) reported always recording and providing patients with the information about teratogenicity and contraception, while 36.6% (n = 41) did so rarely, and 16.1% (n = 18) never documented such details. The summary of the data can be found in Figure 2.

### 3.2. Responses to Nephrologist-Specific Questions

#### 3.2.1. The Assessment of Focus on Women’s Health-Related Issues During Residency Training

According to survey results, reproductive and sex-specific issues in nephrology are addressed inconsistently across residency programs. The influence of sex on the progression of kidney disease was addressed in a limited way according to the majority of respondents (n = 53, 66,2%), while 21.3% (n = 17) stated it was not covered at all. Similarly, the impact of CKD on fertility was also taught in a limited manner, as indicated by 66.3% of respondents (n = 52), with only 7.2% (n = 6) reporting comprehensive coverage.

Fertility of patients on dialysis was slightly better represented, with 36.2% (n = 29) of respondents indicating full coverage, although 56.3% (n = 45) still reported limited inclusion in the residency training. The broader topic of kidney disease and fertility followed a similar pattern, with 66.2% (n = 53) reporting partial coverage and only 25% (n = 20) full coverage.

The impact of immunosuppressive therapy on fertility was the most thoroughly addressed topic, with over half of respondents (n = 41; 51.2%) reporting full coverage and 42.5% (n = 34) indicating limited inclusion.

**Figure 3:**
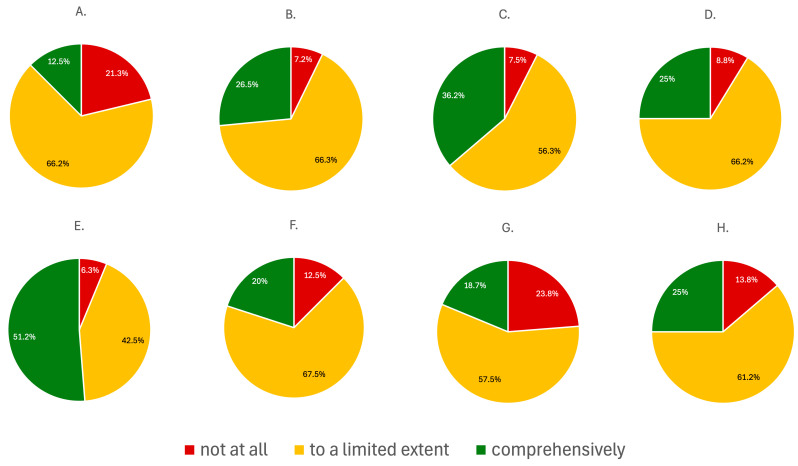
The summary of respondents’ perceptions regarding the coverage of women’s health topics during residency training: (**A**) the impact of gender-related factors on the progression of kidney diseases; (**B**) the impact of CKD on fertility; (**C**) the impact of dialysis on fertility; (**D**) the impact of kidney diseases on fertility; (**E**) the impact of immunosuppressive therapy on fertility; (**F**) the management of women with CKD during pregnancy; (**G**) dialysis during pregnancy; (**H**) kidney diseases specific to pregnancy.

Pregnancy-related nephrology topics were generally underrepresented. Management of pregnant women with CKD was covered in full according to only 20% (n = 16) of respondents, while 67.5% (n = 54) reported limited inclusion into the residency program. Unique aspects of dialysis during pregnancy were the least consistently taught topic, with nearly one in four respondents (n = 19, 23.8%) stating it was not covered at all, and only 18.7% (n = 15) reporting receiving comprehensive training. Similarly, kidney diseases specific to pregnancy were addressed in full according to just 25% (n = 20) of respondents, with 61.2% (n = 49) indicating limited coverage. The summary of the answers is gathered in Figure 3.

#### 3.2.2. Level of the Clinical Experience in the Management of Women’s Health

Nephrology residents and specialists reported varying levels of clinical experience in managing sex-specific and reproductive aspects of CKD. In assessing sex-related differences in CKD progression, 8.8% (n = 7) rated their experience as minimal, 22.5% (n = 18) as low, 30% (n = 24) as moderate, 28.8% (n = 23) as high, and 10.0% (n = 8) as very high. In this field, 44.3% (n = 31) of specialists reported high or very high experience, compared to none among residents; among them, 80% (n = 8) reported minimal or low experience. This difference between these two groups was statistically significant (*p* < 0.001).

Regarding the selection of immunosuppressive therapy in patients of reproductive age, 11.3% (n = 9) reported minimal experience, 21.3% (n = 17) low, 16.3% (n = 13) moderate, 30.0% (n = 24) high, and 21.3% (n = 17) very high. The difference between specialists and residents was also significant in the level of experience in this field (*p* = 0.024), with 55.7% (n = 39) of specialists reporting high or very high experience, while 70% (n = 7) of residents reported minimal or low experience.

In the area of pregnancy planning for women with CKD, 6.3% (n = 5) reported minimal experience, 26.3% (n = 21) low, 26.3% (n = 21) moderate, 23.8% (n = 19) high, and 17.5% (n = 14) very high. It was rated as high or very high by 47.1% (n = 33) of specialists, compared to none among residents. Minimal or low experience was reported by 80% (n = 8) of residents (*p* < 0.001).

When asked about the impact of CKD on fertility, 7.5% (n = 6) reported minimal experience, 15% (n = 12) low, 31.3% (n = 25) moderate, 27.5% (n = 22) high, and 18.8% (n = 15) very high. Around half of specialists (51.4%; n = 36) have high or very high experience, compared to none among residents. Half of the residents (50%, n = 5) reported minimal experience (*p* < 0.001).

The lowest levels of experience both in the specialists and residents groups were reported in the area of assisted reproductive technologies (ART) in women with CKD, with 45% (n = 36) of all answers indicating minimal experience, 30% (n = 24) low, 15% (n = 12) moderate, and only 7.5% (n = 6) and 2.5% (n = 2) measured it as high and very high respectively. All residents (100%, n = 10) reported minimal experience, compared to 37.1% (n = 26) of specialists. Only 11.4% (n = 8) of specialists reported high or very high experience (*p* = 0.001). The summary of the data can be found in Table 4.

#### 3.2.3. The Assessment of the Opinions on the Need to Implement More Women’s Health-Related Issues into the Nephrology Care in Poland

Survey respondents expressed strong support for integrating sex-specific and reproductive health issues into nephrology care and residency training. A majority agreed that kidney disease therapy should be individualized based on patient sex, with 58.8% (n = 47) selecting “rather agree” and 32.5% (n = 26) “strongly agree” on this statement. Only 8.7% (n = 7) disagreed to any extent. The majority of residents (n = 9; 90%) and 90% (n = 63) of specialists agreed to the statement.

The need for establishing referral centers for pregnant women with CKD was affirmed by 58.8% (n = 47) who “strongly agreed” and 27.5% (n = 22) who “rather agreed,” while only 13.7% (n = 11) expressed disagreement. There was no significant difference between residents and specialists on this (n = 8; 80% vs. n = 61; 87,1%).

Collaboration between gynecologists and nephrologists in the care of women with CKD was widely endorsed, with 86.3% (n = 69) “strongly agreeing” and 13.7% (n = 11) “rather agreeing” to the necessity of such cooperation. The 80% (n = 8) of residents and 87.1% (n = 61) of specialists strongly agreeing to the statement.

Regarding reproductive planning, 72.5% (n = 58) “strongly agreed” that renal replacement therapy should be adapted to patients’ procreative intentions, and 25% (n = 20) “rather agreed.” Only 2.5% (n = 2) disagreed, with 90% (n = 9) of residents and 70% (n = 49) of specialists strongly agreeing.

Similarly, 86.3% (n = 69) “strongly agreed”, from which 90% (n = 9) of residents, and 85.7% (n = 60) of specialists, and 13.7% (n = 11) “rather agreed” that immunosuppressive treatment should be individualized based on reproductive plans.

**Figure 4:**
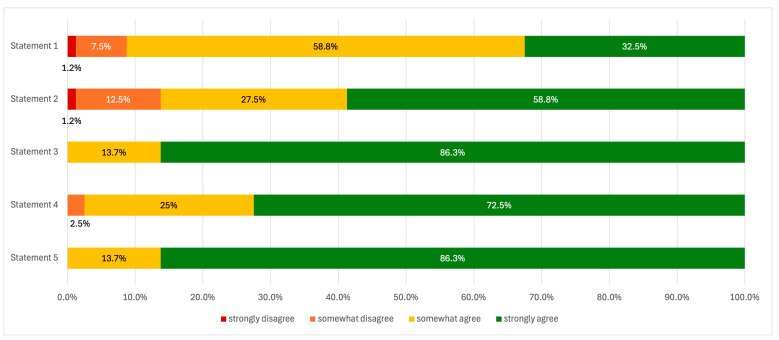
The assessment of the opinions on the need to implement more women’s health-related issues into the nephrology care in Poland: **Statement 1**: It is essential to emphasize the necessity of tailoring kidney disease therapies based on the patient’s gender. **Statement 2:** There is a need to establish reference centers for women with CKD during pregnancy. **Statement 3**: Close collaboration between gynecologists and nephrologists is essential in the management of patients with CKD. **Statement 4**: It is necessary to tailor the form of renal replacement therapy to the procreative plans of the patient. **Statement 5**: The individualization of immunosuppressive therapy in kidney diseases based on the patient’s reproductive plans is essential.

**Figure 5:**
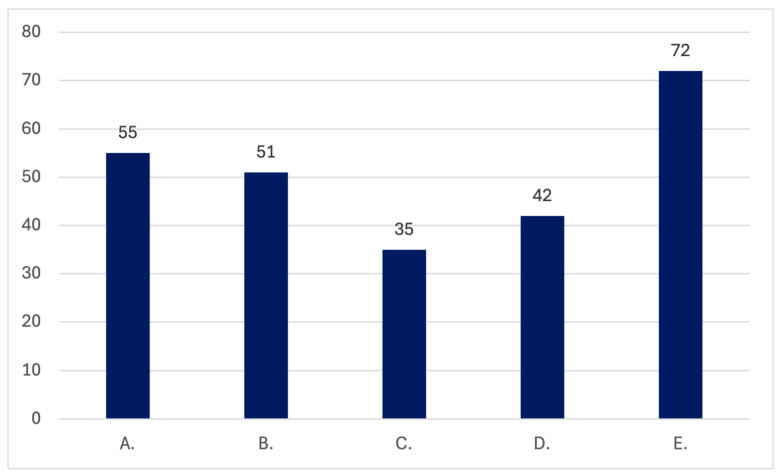
The summary of the preferred educational formats for advancing knowledge on women’s health in kidney disorders (number of respondents): (**A**) conferences; (**B**) webinars; (**C**) mandatory courses during residency; (**D**) additional courses during residency; (**E**) guidelines and recommendations from nephrology and gynecology scientific societies.

In the multiple-choice question, among the 80 respondents who selected at least one answer, the most frequently selected form of training to gain more experience and knowledge on women’s health and kidney diseases was the use of guidelines and recommendations from nephrology and gynecology societies, which was chosen by 72 doctors from this group. Conferences were also a preferred form chosen by 55 respondents, and webinars by 51. Additional specialist courses were selected by 42 participants, while 35 indicated mandatory specialist courses as a desirable format. The data regarding this question is summarized in Figure 4 and Figure 5.

## 4. Discussion

The urgent need to incorporate women’s health and reproductive issues into the management and treatment of patients with kidney disease has been recently extensively recognized and discussed by the Kidney Disease: Improving Global Outcomes (KDIGO) committee [10]. Our survey has followed similar ones performed in various populations of doctors around the world with the shared aim of trying to assess the level of knowledge and confidence among physicians in managing various sex-specific differences in kidney health [13,14,15].

Our results highlight the substantial burden of CKD among patients managed by specialists across various fields. Nearly all respondents (97.4%, n = 113) reported caring for individuals diagnosed with CKD. However, the true prevalence may be even higher, as demonstrated by a recent retrospective epidemiological study conducted in a primary care population. CKD was identified in 14.2% of patients from a high-risk group, based on either eGFR or urinary albumin-to-creatinine ratio (uACR) criteria [16]. These findings underscore the need for heightened diagnostic vigilance, particularly in primary care settings where early detection may be less systematic.

According to our study, the highest levels of self-reported experience in CKD management were observed among physicians working in private practices. In contrast, primary care practitioners generally rated their experience lower, especially regarding sex-specific factors in kidney disease. This observation is partially supported by findings from a Polish survey conducted by Jazienicka-Kiełb et al., which indicated that primary care physicians possess a solid foundational understanding of CKD management, yet expressed a strong interest in further education on the topic [17]. Expanding this educational scope to include sex- and gender-related aspects of kidney health, particularly those relevant to women’s reproductive care, may be a valuable next step in strengthening primary care engagement with nephrology. The strong will of doctors to further expand their knowledge has also been demonstrated in our research.

As revealed in the survey, doctors do not consistently report data relevant to women’s health, such as menstrual history, number of pregnancies, miscarriages, and contraception use. This has also been indicated in other studies conducted among nephrologists in the United States of America (USA) and Canada, which reported that doctors tend to omit taking and reporting a thorough obstetric history, which may lead to missing relevant data on patients’ risk during pregnancy or for future kidney health [13]. However, doctors tend to address procreative plans issues mostly with women, while not always inquiring about these issues with male patients as stated by around 43% of respondents. Still, the common immunosuppressive drug—cyclophosphamide—remaining one of the most potent and commonly used therapeutic agents in the management of various glomerular disorders, poses a significant deleterious impact on both female and male fertility [18].

Another significant issue in the context of women’s reproductive health concerns the use of ACE-I and ARBs. These medications play a well-established role in the management of both hypertension and CKD, as recommended by current clinical guidelines [19,20]. However, due to their known teratogenic effects, ACE-I/ARBs are contraindicated during pregnancy [21]. This presents a significant therapeutic challenge for physicians managing women with CKD who are planning pregnancy or are of childbearing age. Our study revealed that although physicians are generally aware of the risks associated with ACE-I/ARB use during pregnancy, the topic is often overlooked in clinical conversations. Specifically, many respondents reported that they do not routinely address the need for effective contraception when initiating these therapies in women of reproductive age. This gap in communication may reflect time constraints, assumptions about patient awareness, or discomfort discussing reproductive planning while managing patients with kidney disorders. The same conclusions come from the study conducted among the population of women aged 15–45 in the primary care in East London. 81,4% of patients with prescribed ACE-I/ARB had no previous preconception advice recorded [22].

The survey results indicated that both nephrology specialists and residents perceive their training programs as insufficient in preparing them to manage women’s health issues in the context of kidney disorders. Topics related to fertility and pregnancy were reported to be addressed to a limited extent, which was expressed by most respondents. Although, women constitute the majority of patients with CKD [6], the prevalence among women of childbearing age tends to be relatively low—around 3% [23], birth rates in this population are significantly reduced (35.7 children per 1000 person years) compared to non-CKD individuals (46.5 children per 1000 person years) as observed in a population-based study conducted among women in the Stockholm region [24].

The level of expertise in managing female patients with CKD appears to correlate with years of clinical practice, as demonstrated by our survey results. This suggests that clinical confidence in addressing sex-specific aspects of nephrology care is largely acquired through hands-on experience rather than formal training. The limited exposure reported by residents and early-career professionals points to a gap in the current residency program, where topics related to women’s reproductive health in CKD. Introducing these issues earlier in training may significantly enhance both confidence and perceived competence among future nephrologists. Especially, experience in pregnancy-related issues such as pregnancy planning and dialysis during pregnancy was reported to be low, even among specialists. Similar findings were reported in a survey conducted in Germany, where only 40% of nephrologists expressed confidence in managing pregnant patients undergoing dialysis. That study emphasized the urgent need for clearer, evidence-based guidelines to standardize care for pregnant women with CKD and to support clinicians in navigating these complex scenarios [15].

Women with CKD, especially those with already end-stage kidney disease (ESKD), experience various complex issues from hormonal imbalance [25] to sexual dysfunctions [26], which may impair their chances of conceiving naturally. In the event of progress of the assisted reproductive technologies (ART) methods, this procedure becomes a possible solution for this group of patients. Notably, the Italian Society of Nephrology has issued a best practice position statement regarding the various methods of medically associated reproduction. The study group has recognized ART methods as a possible option for women with CKD; however, their opinion is cautious, considering a potential increase in the risk of complications among these patients. The statement has called for personalized counselling with a multidisciplinary healthcare team [27]. To compare, most respondents in our survey have also agreed that a strong collaboration with gynecologists and creating reference centers should become a priority while addressing women’s health in CKD.

It has been revealed in our study that nephrologists express a strong need for further education in the area of women’s health within nephrology. The respondents consider guidelines either developed within nephrology or in collaboration with gynecological societies as a preferred way for addressing these topics and improving clinical competence. To our knowledge Polish Society of Nephrology working group has only addressed the issue of managing pregnancy in CKD; however, other related topics, such as preconception counselling, have not been covered yet. Such comprehensive practice guidelines have already been developed in the Netherlands [28], UK [29], Italy [27,30], or Germany [31].

Similar practice statements and guidelines have also been developed in other fields of medicine. The Working Group of the European Alliance of Associations for Rheumatology (EULAR) has also addressed the need for a clear consensus regarding the use of immunosuppressive medications in male patients and during pregnancy and lactation [32]. Selecting appropriate therapies during these critical periods is also a crucial consideration in the management of patients with glomerular diseases and renal manifestations of other autoimmune disorders. The teratogenic effects and reproductive risks associated with cancer treatment have been extensively discussed by oncology societies, which strongly advocate for comprehensive reproductive counseling. This includes the implementation of fertility preservation strategies such as embryo, oocyte, and ovarian tissue cryopreservation (OTC), ovarian transposition, and conservative gynecologic surgery [33,34]. These options should also be considered when initiating treatment with cyclophosphamide, a drug known for its detrimental impact on fertility. Concurrent administration of gonadotropin-releasing hormone agonists (GnRHa) may help preserve ovarian function and improve the likelihood of pregnancy in the future [35]. Given the growing engagement of the medical community in terms of reproductive health, this calls for an updated version of the Polish guidelines.

Our study obviously was not without limitations. Although it was not possible to determine the exact number of physicians who received the survey, the response rate was relatively low despite broad distribution across various workplace settings. This introduces the possibility of selection bias. The survey included both specialists, residents, and physicians from various fields; this heterogeneity may limit the specificity of conclusions for individual subgroups. However, this diversity reflects real-world clinical practice in Poland, where CKD care is delivered across multiple specialties and different levels of training.

Given the rising CKD and the limited number of nephrologists in Poland, a substantial proportion of patients, particularly those in early stages, are likely to be managed within primary care. Therefore, conducting a similar survey exclusively among primary care physicians would be valuable to more accurately assess their level of expertise and preparedness in addressing women’s health issues in the context of CKD. Such data could inform targeted educational initiatives and promote earlier referral to nephrology services when appropriate [36].

The study mostly relied on self-reported measures of clinical experience using Likert scales. This approach is widely and commonly used in this type of research to assess the perceived level of competence and training needs. However, objective measures, including the exact number of CKD-pregnancy cases managed or the number of reproductive health issues addressed, could strengthen future studies; they were not feasible to collect due to the limited number of voluntary participants.

Despite these limitations, the study offers valuable insights into current practices and unmet educational needs in the management of sex-specific and reproductive health issues in CKD and highlights areas where targeted educational interventions and guideline development may be beneficial.

## 5. Conclusions

To conclude, our study has highlighted a critical discrepancy between the recognized importance of significant sex-specific and reproductive health considerations in the management of CKD and the current level of clinical experience and preparedness to implement these aspects in routine care. Although doctors generally agree on the need for more patient-centered, individualized care and interdisciplinary collaboration, the practical experience in managing female patients with complex issues such as pregnancy planning, renal-replacement therapies during pregnancy, and fertility-related care remains limited. Respondents expressed a clear willingness to obtain more educational tools on this topic. To ensure more equitable care for women with CKD, it seems pivotal to integrate reproductive health issues into nephrology training and develop interdisciplinary guidelines to ensure clear management pathways. By addressing current gaps in this field, it will be possible not only to improve clinical confidence among specialists but also advance the quality of care for a growing population of female patients whose needs for a long time have been unrecognized in nephrology.

## Figures and Tables

**Table 1:** The characteristics of the respondents’ group.

Sex		Number	Percentage
	Female	75	64.66%
	Male	41	35.34%
Years of the clinical experience			
	<5	21	18.10%
	5–10	14	12.07%
	>10	81	69.83%
Stage of professional career			
	Specialist	89	76.72%
	Resident	27	23.28%
Completed or initiated specialization			
	Nephrology	81	69.83%
	Internal medicine	79	68.10%
	Family medicine	4	3.45%
	Cardiology	3	2.59%
	Diabetology	1	0.86%
	Other	36	31.03%
Place(s) of professional employment			
	Multispecialty hospital	84	72.41%
	District/municipal hospital	41	35.34%
	Specialist outpatient practice	32	27.59%
	Primary healthcare practice	16	13.79%
	Non-public (private) facility	8	6.90%

**Table 2:** The characteristics of the patient population managed by the survey respondents.

Predominant Age Group of Your Patient		Number	Percentage
	18–40	5	4.31%
	40–60	19	16.38%
	60–80	89	76.72%
	>80	3	2.59%
Estimated predominant sex of patients			
	More or less the same	65	56.04%
	Majority of women	25	21.55%
	Majority of men	12	10.34%
	Difficult to estimate	14	12.07%
Estimated predominant education level of patients			
	Secondary/vocational education	98	88.29%
	Primary education	13	72.22%
	Higher education	5	4.31%
Estimated predominant form of professional activity of patients under care			
	Retired/pensioner	94	83.93%
	Professionally active	19	16.52%
	Student	2	9.09%
	Unemployed	1	0.85%
Patients diagnosed with CKD			
	Yes	113	97.41%
	No	3	2.59%
Estimated percentage of patients with the diagnosis of CKD			
	<10%	12	10.62%
	10–50%	31	27.43%
	>50%	70	61.95%
Patients undergoing dialysis under care			
	Yes	98	84.48%
	No	18	15.52%
Predominant form of dialysis therapy among the patients under care			
	Hemodialysis	85	86.73%
	Peritoneal dialysis	4	4.08%
	More or less the same percentage	9	9.18%
Pregnant patients under your care			
	Yes	44	37.93%
	No	72	62.07%
Estimated number of pregnant patients under care annually			
	<50/year	39	88.64%
	50–100/year	4	9.09%
	>100/year	1	2.27%

**Table 3:** The summary of the respondents’ level of clinical experience in the management of patients with CKD across various specialties: 1 (very low experience), 2 (low experience), 3 (moderate experience), 4 (high experience), 5 (very high experience).

Question 1: Managing patients with CKD.					
	1	2	3	4	5
Number	10	5	17	16	68
Percentage	8.62%	4.31%	14.66%	13.79%	58.62%
Question 2: The impact of gender-related factors on the development of kidney diseases.					
	1	2	3	4	5
Number	23	27	23	23	20
Percentage	19.83%	23.28%	19.83%	19.83%	17.24%
Question 3: Kidney diseases related to pregnancy.					
	1	2	3	4	5
Number	39	27	26	17	7
Percentage	33.62%	23.28%	22.41%	14.66%	6.03%
Question 4: The impact of immunosuppressive therapy on fertility.					
	1	2	3	4	5
Number	32	19	26	19	20
Percentage	27.59%	16.38%	22.41%	16.38%	17.24%

**Table 4:** Summary of the nephrologists’ level of clinical experience in management of women’s health: 1 (very low experience), 2 (low experience), 3 (moderate experience), 4 (high experience), 5 (very high experience).

Question 1: Differences in the course and progression of CKD based on the patient’s gender.					
	1	2	3	4	5
Number	7	18	24	23	8
Percentage	8.75%	22.50%	30.00%	28.75%	10.00%
Question 2: Appropriate selection of immunosuppressive therapy for individuals of reproductive age.					
	1	2	3	4	5
Number	9	17	13	24	17
Percentage	11.25%	21.25%	16.25%	30.00%	21.25%
Question 3: Planning pregnancy in patients with CKD.					
	1	2	3	4	5
Number	5	21	21	19	14
Percentage	6.25%	26.25%	26.25%	23.75%	17.50%
Question 4: Differences in dialysis management during pregnancy.					
	1	2	3	4	5
Number	18	17	20	11	14
Percentage	22.50%	21.25%	25.00%	13.75%	17.50%
Question 5: The impact of CKD on fertility.					
	1	2	3	4	5
Number	6	12	25	22	15
Percentage	7.50%	15.00%	31.25%	27.50%	18.75%
Question 6: Assisted reproductive technologies (ART) in women with CKD.					
	1	2	3	4	5
Number	36	24	12	6	2
Percentage	45.00%	30.00%	15.00%	7.50%	2.50%

## Data Availability

The original contributions presented in this study are included in the article. Further inquiries can be directed to the corresponding author.

## Supplementary Materials

The following supporting information can be downloaded at: https://www.mdpi.com/article/10.3390/jcm15010196/s1.

## Abbreviations

The following abbreviations are used in this manuscript:

ACE-I/ARBs	angiotensin-converting enzyme inhibitors or angiotensin II receptor blockers
ART	assisted reproductive technologies
CKD	chronic kidney disease
eGFR	estimated glomerular filtration rate
EULAR	European Alliance of Associations for Rheumatology
GnRHa	gonadotropin-releasing hormone agonists
HELIUS	Healthy Life in an Urban Setting
KDIGO	Kidney Disease: Improving Global Outcomes
MMG	mammography
NCD	noncommunicable disease
OTC	ovarian tissue cryopreservation
uACR	urinary albumin-to-creatinine ratio
USA	the United States of America
USG	ultrasound
WHO	World Health Organisation
