# Supplementary material for: Toward Equitable Kidney Care: Insights from the Survey Among Polish Doctors on the Women’s Health in Chronic Kidney Disease Management
Source: J Clin Med. 2025 Dec 26;15(1):196. doi: 10.3390/jcm15010196 (PMC12786750; doi:10.3390/jcm15010196)
Supplement: Supplementary file 1 [file jcm-15-00196-s001.zip › jcm-4041328-supplementary.pdf]

Dear Sir/Madam,

The global population is witnessing a steadily increasing incidence of chronic kidney disease (CKD). As a result, patients affected by this condition are likely to visit specialists more frequently. Statistics show that women are more prone to CKD than men; however, current guidelines lack detailed recommendations for managing patients based on their gender.

The purpose of our survey is to explore your experiences in working with female and male patients suffering from CKD, with a particular focus on women's health-related issues.

Completing the survey will take approximately 10 minutes. The survey is entirely anonymous. By filling out the survey, participants consent to the use of anonymized data in the research described above.

We greatly appreciate your time spent filling out the survey.

The team of the Department of Nephrology, Dialysis Therapy, and Internal Medicine at the Medical University of Warsaw, under the leadership of Professor Jolanta Małyszko, MD, PhD.

First part

1. Sex:
   - female
   - male
2. How many years of the clinical experience you have:
   - <5 years
   - 5-10 years
   - >10 years
3. Please indicate your stage of professional career:
   - specialist
   - resident doctor - early resident (first two years)
   - resident doctor - at the end of the training (more than two years of training completed)
   - intern doctor
4. Please select the specialization you have completed or started:
   - nephrology
   - cardiology
   - diabetology
   - family medicine
   - internal medicine
   - other
5. Please indicate your place(s) of professional emloyment (multiple-choice question):
   - multispecialty hospital
   - district/municipal hospital
   - specialist outpatient practice
   - primary healthcare practice

- non-public (private) facility

6. What is the predominant age group of your patient:
   - 18-40
   - 40-60
   - 60-80
   - >80
7. What is the estimated predominant gender of your patients:
   - majority of women
   - majority of men
   - more or less the same
   - diNicult to estimate
8. What is the estimated predominant education level of your patients:
   - primary education
   - secondary/vocational education
   - higher education
9. What is the estimated predominant form of professional activity of patients under your care:
   - student
   - professionally active
   - unemployed
   - retired/pensioner
10. Do you have patients diagnosed with CKD (chronic kidney disease) under your care?
    - Yes.
    - No.
11. If you answered 'yes' to the previous question what is the estimated percent of your patients with the diagnosis of CKD:
    - <10%
    - 10-50%
    - >50%
12. Do you have patients undergoing dialysis under your care?
    - Yes.
    - No.
13. If you answered 'yes' to the previous question please specify the predominant form of dialysis therapy among the patients under your care:
    - hemodialysis
    - peritoneal dialysis
    - more or less the same percentage
14. Do you have pregnant patients under your care?
    - Yes.
    - No.

15. If you answered ‘yes’ to the previous question please estimate the number of pregnant patients under your care annually:
- o <50 a year rocznie
- o 50-100 a year rocznie
- o >100 a year

16. Please rate your level of experience and knowledge of the following topics according to the scale below (1 - very low experience, 2 - low experience, 3 - moderate experience, 4 - high experience, 5 - extensive, very high clinical experience):

	**1**	**2**	**3**	**4**	**5**
Managing patients with chronic kidney disease (CKD).					
The impact of gender-related factors on the development of kidney diseases.					
Kidney diseases related to pregnancy.					
The impact of immunosuppressive therapy on fertility.					

17. Please estimate how often you inquire about the following issues when taking a medical history from female patients:

	**Never**	**Rarely**	**Often**	**Always**
Menstrual cycle regularity.				
The number of pregnancies.				
The number of miscarriages.				
Vasomotor symptoms during the perimenopausal period.				
Contraception methods.				
Date of the last gynecological visit.				
Date of the last mammography/ultrasound breast examination.				
Date of the last Pap smear test.				

18. Do you inquire about procreative plans from individuals of reproductive age?:
- o only women
- o only men
- o yes, both women and men
- o no

19. Do you use ACE inhibitors (ACE-I) or angiotensin receptor blockers (ARB) in your practice?:

- ○ yes
- ○ no

20. If you anwered 'yes' to the previous question please specify the indications for which you prescribe them (multiple-choice question):

- ○ hypertension
- ○ heart failure/cardioprotection
- ○ nephroprotection
- ○ other

21. Before initiating treatment with ACE inhibitors (ACE-I) or angiotensin receptor blockers (ARB):

	**Never**	**Rarely**	**Often**	**Always**
do you inquire about procreative plans?				
do you inform patients about the potential teratogenic eVects of the medications?				
do you inform patients about the necessity of using eVective contraception during the course of the therapy?				
do you record in medical documentation the fact that you informed patients about the teratogenic eVects and the necessity of using eVective contraception?				

Second part – <u>questions only for nephrology specialists and residents</u>

1. In your opinion, were/are the following topics adequately addressed during specialization training?:

	Not at all	To a limited extent	Comprehensively
The impact of gender-related factors on the progression of kidney diseases.			
The impact of chronic kidney disease (CKD) on fertility.			
The impact of dialysis therapy on fertility.			
The impact of kidney diseases on fertility.			
The impact of immunosuppressive therapy on fertility.			
The management of women with chronic kidney disease (CKD) during pregnancy.			
Dialysis during pregnancy.			
Kidney diseases specific to pregnancy.			

2. Please rate your level of experience and knowledge of the following topics according to the scale below (1 - very low experience, 2 - low experience, 3 - moderate experience, 4 - high experience, 5 - extensive, very high clinical experience):

	1	2	3	4	5
DiVerences in the course and progression of chronic kidney disease (CKD) based on the patient's gender.					
Appropriate selection of immunosuppressive therapy for individuals of reproductive age.					
Planning pregnancy in patients with chronic kidney disease (CKD).					
DiVerences in dialysis management during pregnancy.					
The impact of chronic kidney disease (CKD) on fertility.					
Assisted reproductive technologies (ART) in women with chronic kidney disease (CKD).					

3. Please assess whether you agree with the following statements::

	**Strongly disagree**	**Somewhat disagree**	**Somewhat agree**	**Strongly agree**
It is essential to emphasize the necessity of tailoring kidney disease therapies based on the patient's gender.				
There is a need to establish reference centers for women with chronic kidney disease (CKD) during pregnancy.				
Close collaboration between gynecologists and nephrologists is essential in the management of patients with chronic kidney disease (CKD).				
It is necessary to tailor the form of renal replacement therapy to the procreative plans of the patient.				
The individualization of immunosuppressive therapy in kidney diseases based on the patient's reproductive plans is essential.				

4. Please select which forms of training you believe would be useful to deepen knowledge about women's health and kidney diseases (multiple-choice question):

- Conferences
- Webinars
- Mandatory courses during residency training
- Additional courses during residency training
- Guidelines and recommendations from nephrology and gynecology scientific societies
